# Sustained postsynaptic kainate receptor activation downregulates AMPA receptor surface expression and induces hippocampal LTD

**DOI:** 10.1016/j.isci.2025.112152

**Published:** 2025-03-05

**Authors:** Jithin D. Nair, Ellen Braksator, Busra P. Yucel, Alexandra Fletcher-Jones, Richard Seager, Jack R. Mellor, Zafar I. Bashir, Kevin A. Wilkinson, Jeremy M. Henley

## Main text

(iScience *24*, 103029; September 24, 2021)

In the originally published version of this article, the GAPDH loading blot was inadvertently duplicated in Figures 1Bi, 3A, 4A, 5Ai, 5Bi, and 5Ci. After reviewing the raw data, we provide the correct figures below. These changes do not affect the scientific conclusions of the paper. The authors apologize for any inconvenience and confirm these updates.

**Figure:**
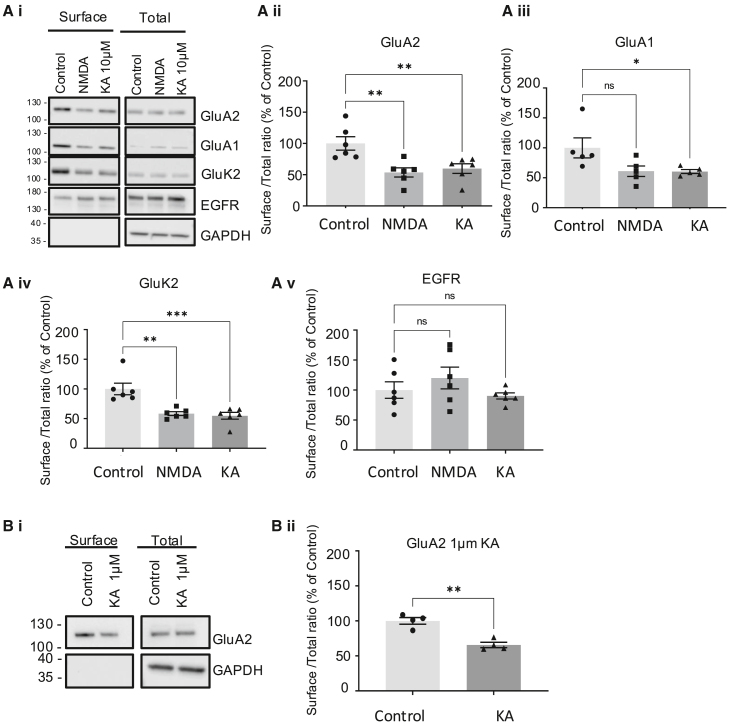
Figure 1. KAR activation reduces surface expression of AMPARs and KARs

**Figure:**
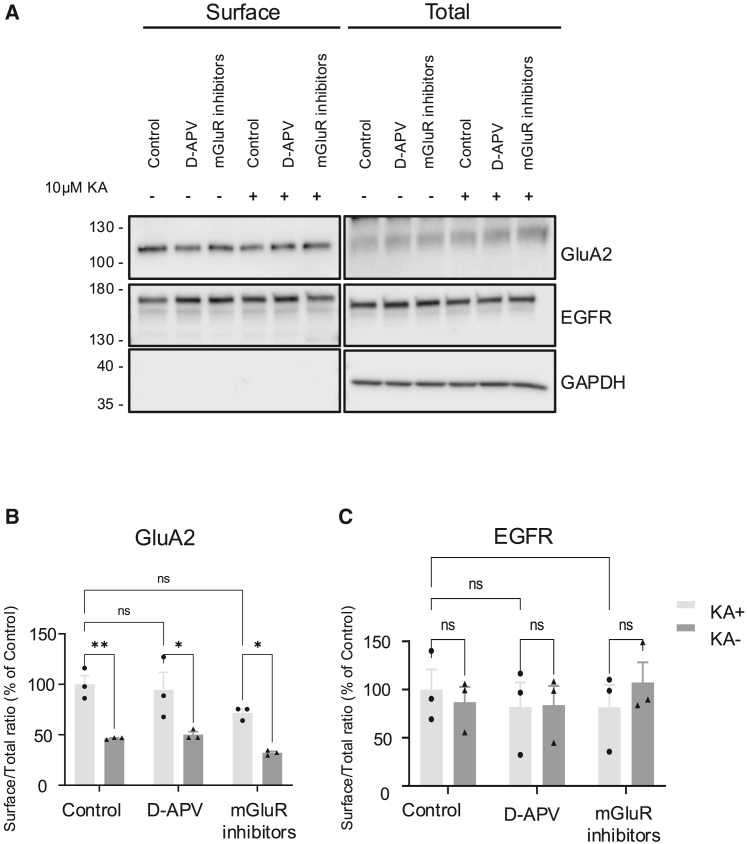
Figure 3. KA-induced decreases in AMPAR surface expression are independent of NMDAR or mGluR1/mGluR5 activation

**Figure:**
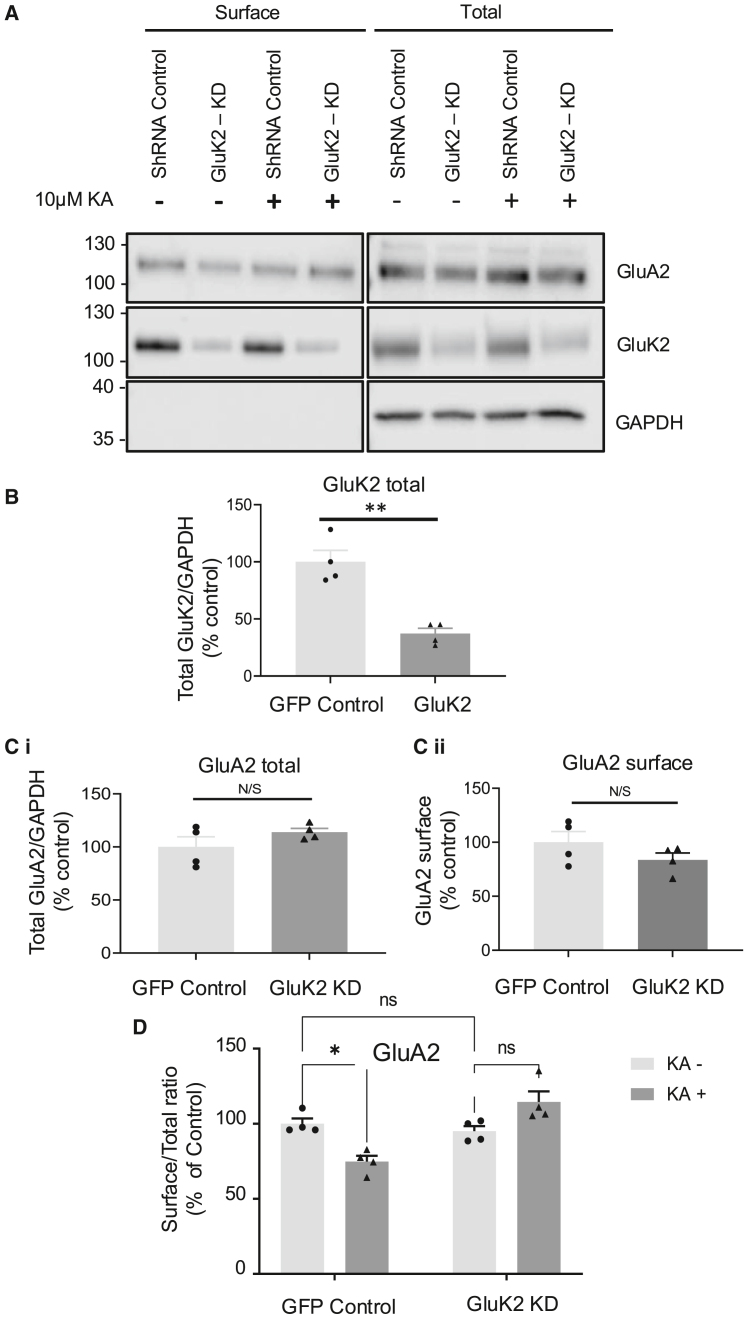
Figure 4. GluK2-containing KARs mediate the reduction in surface expression of AMPARs

**Figure:**
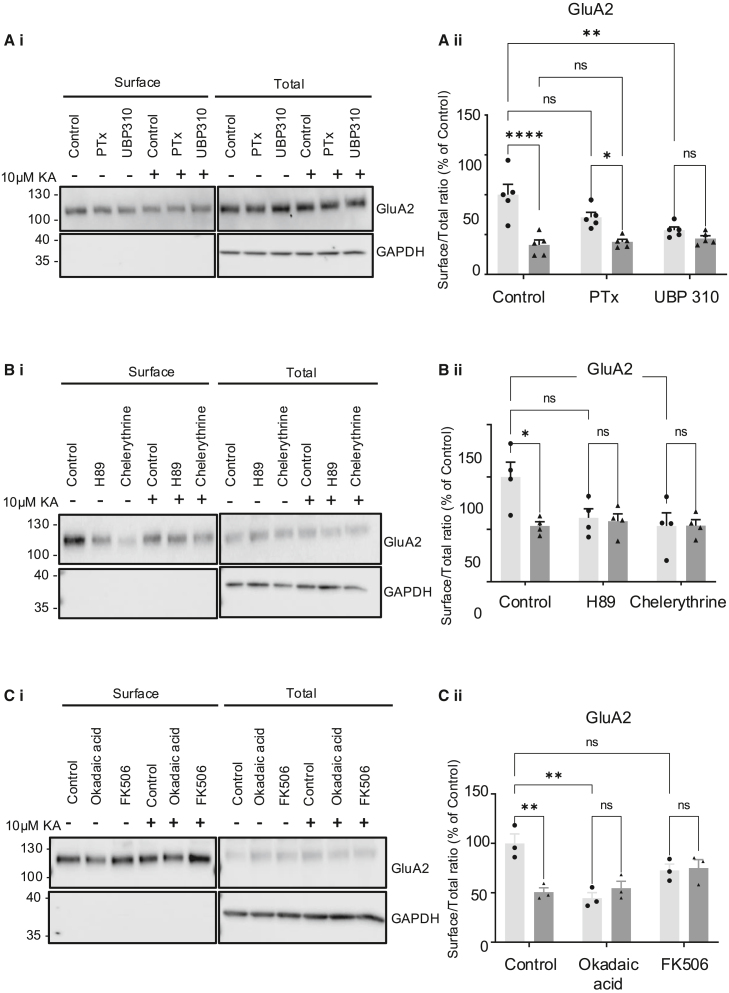
Figure 5. KA-induced decreases in AMPAR surface expression require ionotropic KAR signaling, PKA, and PKC

